# Changes in visual acuity and corneal aberrations after presbyopia correction using micro-monovision multifocal bi-aspheric ablation: six-month results

**DOI:** 10.1186/s12886-026-04741-9

**Published:** 2026-03-25

**Authors:** Xiaofan Wang, Li Li, Yinuo Lin, Peng Ji, Yifan Tang, Suqi Xian, Hongsheng Bi

**Affiliations:** 1 Shandong University of Traditional Chinese Medicine, Jinan, Shandong China; 2 Shandong Clinical Medicine Research Center, Affiliated Eye Hospital of Shandong University of Traditional Chinese Medicine, No. 48 Yingxiongshan Road, Shizhong District, Jinan City, Shandong Province China

**Keywords:** Presbyopia, Multifocal, Corneal aberrations, PresbyMAX

## Abstract

**Aim:**

To assess visual outcomes and corneal aberration changes following micro-monovision multifocal bi-aspheric ablation (PresbyMax monocular) for presbyopia correction.

**Method:**

25 patients (50 eyes) underwent PresbyMAX monocular treatment. Pre and postoperative evaluations (1 week, 1, 3, and 6 months) included uncorrected distance (UDVA), intermediate (UIVA), near (UNVA), and corrected distance visual acuity (CDVA). Corneal aberration and Strehl ratio (SR) were analyzed within 3-mm and 6-mm zones. The National Eye Institute 25-Item Visual Function Questionnaire (NEI-VFQ-25) was used to assess visual function preoperatively and at 6 months postoperatively.

**Result:**

At 6 months, binocular UDVA and UIVA were 0 (− 0.1, 0) logMAR and − 0.1 (− 0.1, 0) logRAD, respectively. Spectacle independence reached 96%, with 96%, 100%, and 96% achieving UDVA ≥ 0.1 logMAR, UIVA ≥ 0.1 logRAD, and UNVA ≥J1. No eyes lost corrected distance visual acuity. In non-dominant eyes, 3-mm corneal zones exhibited negative shifts in fourth-order spherical aberration (${Z}_{4}^{0}$) and sixth-order spherical aberration (${Z}_{6}^{0}$) (both *p* < 0.001). In 6-mm corneal zones, ${Z}_{4}^{0}$ exhibited no significant change; however, compared to the dominant eye, it was significantly more negative (*p* < 0.001). ${Z}_{6}^{0}$ increased positively with a statistically significant difference (*p* < 0.001). NEI-VFQ-25 scores improved from 74.63 (71.70, 81.81) to 91.15 (85.23, 93.08) (*p* < 0.001).

**Conclusion:**

The PresbyMAX protocol enhances near vision by strategically manipulating aberrations to increase depth of focus in the non-dominant eye, while the dominant eye maintains distance and intermediate vision. This synergistic binocular approach achieves satisfactory full-range vision, resulting in a 96% spectacle independence rate and high patient satisfaction.

**Clinical trial registration number:**

ChiCTR2300074821, registration date 2023-08-17.

## Introduction

Presbyopia is an age-related physiological condition characterized by a progressive decline in the eye’s accommodative ability, primarily due to reduced lens elasticity and ciliary muscle function [1]. The global population with presbyopia is projected to exceed 2 billion by 2030 [2]. Uncorrected and inadequately corrected presbyopia will have an impact on social and economic development. Surgical correction of presbyopia has increasingly become a primary focus of ophthalmic research [2].

The correction of presbyopia is mainly divided into two types: (1) Dynamic approach: It aims to restore the self-accommodation ability, such as accommodating intraocular lenses, scleral expansion, etc.; (2) Static approach: By increasing the depth-of-focus (DoF), it can relieve symptoms and improve near visual acuity, such as monovision, multifocal intraocular lenses, etc [3]. However, currently, the clinical treatment methods of dynamic correction are still in the exploratory stage [3].

The approaches of refractive surgery for correcting presbyopia can be divided into corneal surgery and lens surgery. In comparison, lens surgery is more suitable for patients with lens opacification accompanied by presbyopia. However, it has risks such as intraocular infection. On the other hand, corneal surgery is less invasive and is more suitable for patients with transparent lenses but still troubled by presbyopia [4]. Corneal surgeries include monovision, PresbyLASIK, inlays, etc. The monovision scheme is prone to cause phenomena such as difficulty in tolerance and loss of intermediate visual acuity due to large anisometropia, and it is more suitable for patients in the early stage of presbyopia [5]. Inlay is a surgery that makes incisions in the peripheral cornea and places a lens to change the corneal refractive power to treat mild refractive errors accompanied by presbyopia. It uses the principle of pinhole design or creating corneal multifocality and other methods to improve near visual acuity. However, there are still phenomena such as postoperative glare, corneal opacity, and decline of CDVA [6].

With the rapid development of refractive surgery, excimer lasers has become the mainstream method for correcting various types of refractive errors due to its safety and effectiveness [7]. On this basis, Ruiz [8] proposed to reshaped the cornea into a multifocal profile to increase the DoF and improve near vision, that is, PresbyLASIK [5, 9]. According to the differences in the near-vision area and the multifocal ablation mode, PresbyLASIK can be divided into the peripheral near-vision pattern (central distance vision and peripheral near vision) and the central near-vision pattern (central near vision and peripheral distance vision). Since the central near-vision pattern is more sparing of the cornea and conforms to the physiological state of the human eye when looking at near objects, the central type is currently more commonly used [9]. PresbyMAX is a commonly used central PresbyLASIK in clinical practice. It combines the advantages of micro-monovision, the multifocal mode, and the bitoric aspheric design, enabling patients to obtain clear full-range vision [10].

Although this surgery can improve patients’ distance, intermediate, and near vision, its acceptance rate is still lower than that of the monovision scheme, and there is a lack of medium- and long-term follow-up studies after the operation. In this study, by observing and following up on indicators such as the patients’ visual acuity, aberrations, and visual quality six months after the operation, we evaluated the changes in the patients’ visual acuity and corneal aberrations after the surgery.

## Methods

### Patients

This study is a prospective self-controlled trial, and 25 patients (50 eyes) who underwent PresbyMAX monocular correction for presbyopia at the Affiliated Eye Hospital of Shandong University of Traditional Chinese Medicine from January 2023 to December 2024 were enrolled. It has been approved by the Ethics Committee of the Affiliated Eye Hospital of Shandong University of Traditional Chinese Medicine (HEC-KS-2020003KY05) and strictly adheres to the Declaration of Helsinki. All patients have signed the informed consent form.

All patients met the following inclusion criteria: aged 40 ~ 55, the range of spherical refraction was from − 8 diopters (D) to -0.5 D, and the range of cylindrical refraction was from − 4.00 D to 0 D; the near addition (ADD) + 1.25 ~ + 2.50D; the CDVA was better than 0.1 logMAR, and the best corrected near visual acuity(CNVA) was better than J2; in the monovision test, the binoculars could tolerate at least 1 D of anisometropia; the photopic pupil diameter was between 2.5 and 3.5 mm, and the scotopic pupil diameter was greater than 4.5 mm, the spherical aberration (SA) at a corneal diameter of 6 mm > 0.0 μm; and the higher - order aberrations (HOAs) was less than 0.5 μm; the preoperative anterior corneal surface curvature is 40.0 ~ 48.0D, and the postoperative anterior corneal surface curvature is 35.0D ~ 48.0D. The exclusion criteria were as follows: suffering from corneal diseases or ocular infections; abnormalities in preoperative corneal topography and biomechanical testing, the central corneal thickness was less than 480 μm, or the thickness of the corneal stroma after ablation was less than 300 μm; pupil offset ≥ 0.7 mm; having other eye diseases affecting vision except refractive errors, a history of ophthalmic surgeries (including laser corneal refractive surgeries), a history of trauma, etc., having overly high expectations for the surgery, or being patients who were engaged in excessive close work.

### Examination

Preoperative examinations included subjective and objective visual acuity, intraocular pressure measurement (NT-510, Nidek, Japan), Sirius three-dimensional anterior segment analyzer examination(Sirius system, C.S.O, Italy), Master IOL measurement (IOL Master 500, Zeiss, German), Pentacam corneal topographer examination (Pentacam^®^ HR, Oculus, Germany), Corvis biomechanical examination, anterior segment (CORVIS ST, Oculus, Germany) and fundus examinations, visual function examination (preliminary measurement of the patient’s ADD), and contrast sensitivity test. The dominant eye was identified with a pinhole card by having the patient view a distant target through its central aperture with both arms extended. The eye that maintained a clear view of the target when each eye was alternately occluded was designated as the dominant eye. When the results of multiple measurements were consistent, the dominant eye was determined, and soft contact lenses were fitted to determine the appropriate ADD and anisometropia degree.

At 1 week, 1 month, 3 months, and 6 months after the surgery, follow-up examinations were conducted to assess the UDVA, UIVA, UNVA, as well as the CDVA of the dominant eye, non-dominant eye, and both eyes. Subjective vision acuity was performed. The Sirius analyzer was used to measure the aberrations and SR of the cornea within the diameter ranges of 3 mm and 6 mm. At pre-operatively and 6 months postoperatively, the NEI-VFQ-25 was used to investigate the patients’ satisfaction with postoperative visual acuity and visual quality [11].

The distance visual acuity (at 5 m) was assessed using a standard logarithmic chart and expressed in logMAR visual acuity; the intermediate visual acuity was measured with a standard logarithmic intermediate/near chart expressed in logRAD (at 80 cm); and the near visual acuity was expressed in Jaeger visual acuity (at 40 cm). The unit of refractive power was Diopter (D), and the unit of aberration was also D.

### Surgical procedure

All surgeries were performed by an experienced physician. The VisuMax femtosecond laser system (Carl Zeiss VisuMax, Germany) was used to create a corneal flap with a thickness of 100 μm, a diameter of 8.1 mm, and a hinge angle of 90°. The SCHWIND AMARIS 1050RS excimer laser machine (Schwind eye-techsolutions GmbH, Germany) was applied for corneal stromal ablation. For the dominant eye, the Aberration-Free mode was selected, and the target refractive power was set at 0. For the non-dominant eye, the PresbyMAX monocular ablation mode was chosen, with an optical zone range of 6.2–6.8 mm. According to the manufacturer’s guidelines, a near addition(ADD) within the range of + 1.25-+2.50 D was introduced at the central area of 3 mm (usually set as + 1.75 D. For myopic patients, for every − 1.0 D of the equivalent spherical value, the ADD was adjusted upward by 0.05 D), and the target refractive power was − 0.89 D.

After the treatment, the stroma was irrigated with balanced salt solution, the corneal flap was repositioned, and 0.3% Tobramycin and Dexamethasone Eye Drops were instilled. The repositioning of the cornea was observed under the slit lamp.

### Statistical analyses

Statistical analysis was performed using IBM Statistics SPSS 25.0 (v.25, IBM Corporation). The normality of the data was tested by the Shapiro—Wilk test. For the data with a normal distribution, they were expressed as the mean ± standard deviation, and paired t-test or one-way analysis of variance was used; for the data with a non-normal distribution, they were expressed as the median and interquartile range, and the Friedman Test or the Wilcoxon signed-rank test was applied. A *p* value less than 0.05 was considered to indicate a statistically significant difference.

## Result

A total of 25 patients (50 eyes) were enrolled in this study. All patients met the inclusion and exclusion criteria. The average age of the patients before the operation was 47.48 (± 3.63) years. The sphere of the dominant eye and the non-dominant eye was − 4.01 ± 1.91D, -3.84 ± 1.88D, respectively, the cylindrical powers of them are − 0.72 ± 0.52D, -0.79 ± 0.51D, respectively, and the equivalent spherical (SE) of them are − 4.38 ± 1.97D, -4.24 ± 1.87D, respectively. The preoperative baseline characteristics of the patients are presented in Table 1.

**Table 1:** Patient characteristics

Parameter	DE	NE
Eyes	25/25	25/25
Age	47.48(± 3.63)	
Sphere(D)	-4.02 ± 1.95	-3.84 ± 1.92
Cylinder(D)	-0.72 ± 0.53	-0.79 ± 0.52
SE (D)	-4.38 ± 2.01	-4.24 ± 1.91
UDVA (logMAR)	1.2(1.1~1.3)	1.2(1~1.3)
UIVA (logMAR)	0.4(0.3~0.5)	0.4(0.~0.5)
UNVA (Jaeger)	J3~J13	J2 ~ J11
Z(4,0) (3 mm) (µm)	0.05(0.03~0.06)	0.05(0.02~0.08)
Z(4,0) (6 mm) (µm)	0.18(0.15 0.21)	0.16(0.13~0.2)
Z(6,0) (3 mm) (µm)	0(-0.01~0)	0(-0.04~ 0.01)
Z(6,0) (6 mm) (µm)	0(-0.01~0)	0(-0.01~0.01)
Planned ADD(D)		1.94 ± 0.092

DE = dominant eye; NE = non-dominant eye; SE = spherical equivalent

### Efficacy

**Fig. 1:**
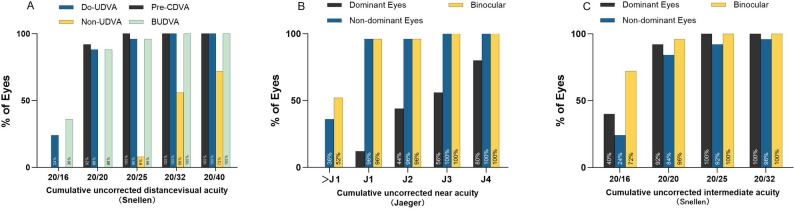
**A**: Cumulative UDVA 6 months after surgery. **B**: Cumulative UIVA 6 months after surgery. C: Cumulative UNVA 6 months after surgery. Do-UDVA= UDVA of dominant eyes. Non-UDVA=UDVA of non-dominant eyes

At 6 months postoperatively, the mean UDVA of the dominant eye, non-dominant eye, and both eyes was 0 (-0.05 to 0) logMAR, 0.2 (0.2 to 0.4) logMAR, and 0 (-0.1 to 0) logMAR, respectively. A statistically significant difference was observed between pre- and postoperative assessments for both eyes (*p* < 0.001). At 6 months postoperatively, 96% of both the dominant eyes and binocular eyes achieved an UDVA of 0.1 logMAR or better, and 88% of the eyes reached 0 logMAR or better. For the non-dominant eyes, approximately 56% of the operated eyes reached 0.2 logMAR or better (Fig. 1. A).

The UIVA of the dominant eye, non-dominant eye, and the binocular uncorrected intermediate visual acuity (BUIVA) were − 0.1 (-0.1 to 0) logRAD, 0 (-0.05 to 0) logRAD, and − 0.1 (-0.1 to 0) logRAD, respectively (*p* < 0.001). At 6 months postoperatively, the dominant eye was significantly better than the non-dominant eye (*p* < 0.05). After the surgery, the UIVA of the dominant eye and binocular eyes of all patients reached 0.1 logRAD or better, and approximately 92% of the non-dominant eyes had UIVA reaching 0.1 logRAD or better (Fig. 1. C).

Only 28% of eyes could achieve J3 in UNVA before surgery. Six months after surgery, the UNVA of 96% of both eyes and non-dominant eyes reached J1 or better. The UNVA of the dominant eye of 80% of the patients reached J5 or better, and 44% reached J2 or better (Fig. 1. B).

### Safety

**Fig. 2:**
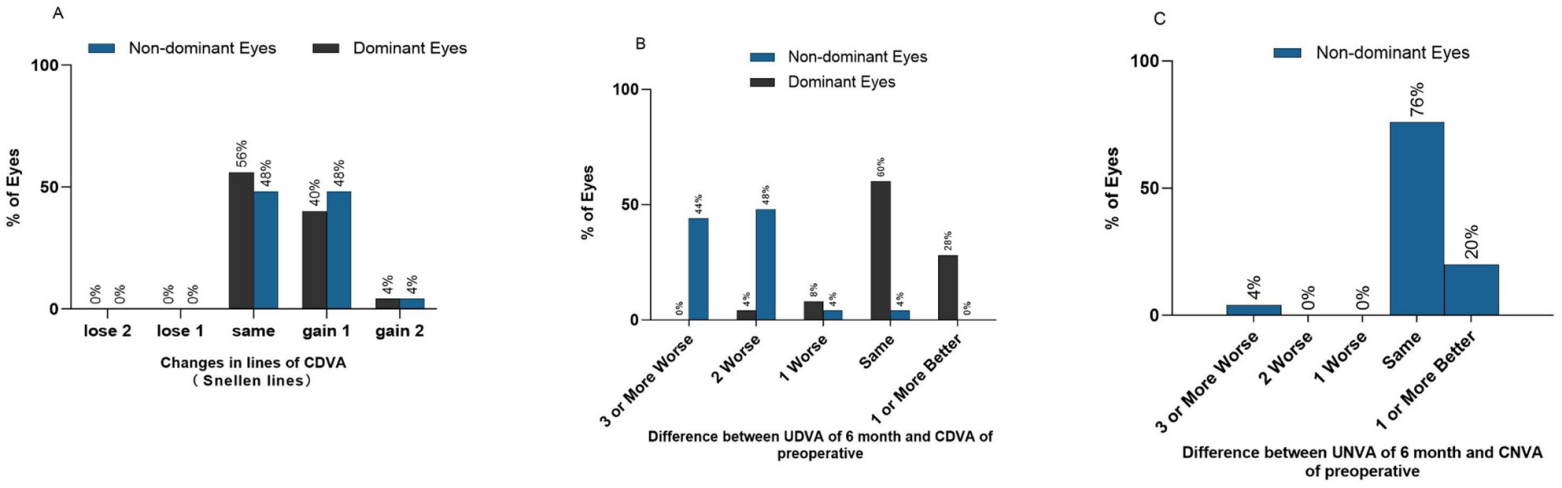
**A**: Change in Snellen line of CDVA. **B**: Difference between UDVA of 6 month and CDVA of preoperative. **C**: Difference between UNVA of 6 month and CNVA of preoperative

At 3 months postoperatively, the CDVA of 20% (10 eyes) of the operated eyes gained one line, 78% (39 eyes) remained unchanged, and 2% (1 eye) lost one line. There were no cases of CDVA increasing or decreasing by two lines. At 6 months postoperatively, the CDVA of 4% (2 eyes) of the dominant eyes gained two lines, 42% (21 eyes) gained one line, 56% (28 eyes) remained unchanged, and there were no cases of CDVA loss (Fig. 2. A).

### Predictability

**Fig. 3:**
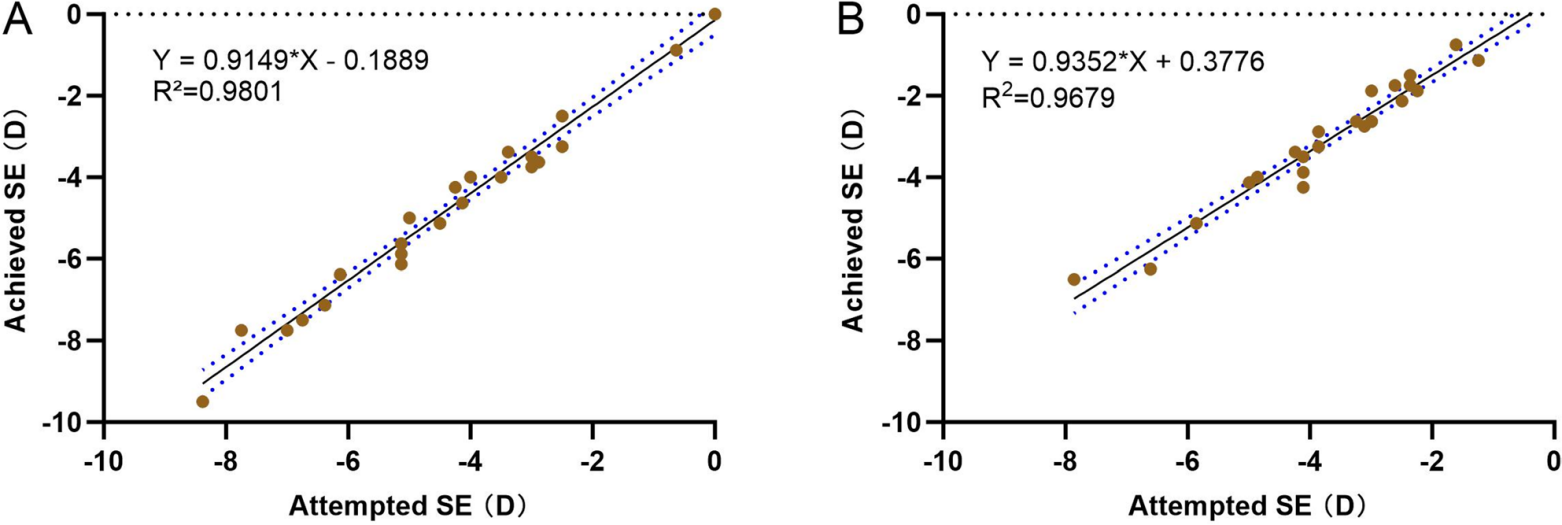
**A**: Attempted refraction vs. the achieved SE refraction in the dominant eye. **B**: Attempted SE refraction vs. the achieved SE refraction in the nondominant eye. SE = spherical equivalent

The target refraction was set at 0 D for the dominant eye and at -0.89 D for the non-dominant eye. The predictability of the SE correction at 6 months is detailed in Fig. 3. The attempted and achieved astigmatic corrections were closely matched, with the dominant eye(R^2^ = 0.9801) showing marginally better predictability than the non-dominant eye(R^2^ = 0.9679).

### Accuracy

**Fig. 4:**
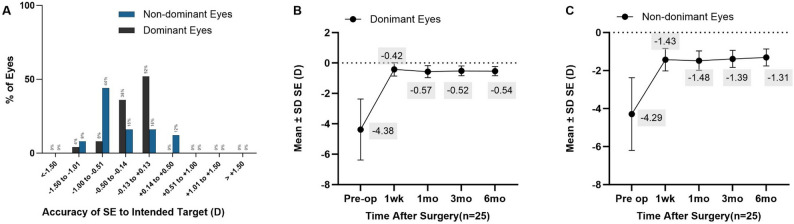
**A**: Accuracy of SE to intended target. **B**: Stability of spherical equivalent refraction (dominant eyes). **C**: Stability of spherical equivalent refraction (non-dominant eyes)

The accuracy was shown in Fig. 4.A. For the dominant and non-dominant eyes, 96% and 92% were within ± 1.00 D of the target refraction, respectively; 88% and 44% were within ± 0.50 D. All eyes were within ± 1.50 D of the intended target.

### Stability

The SE of the dominant eye and non-dominant eye before surgery was − 4.38 ± 1.97D and − 4.24 ± 1.87D, respectively, and at 6 months after surgery, they were − 0.54 ± 0.3D and − 1.31 ± 0.45D, respectively, with significant differences before and after surgery. The postoperative SE was relatively stable, and there was no statistically significant difference at each time point (Fig. 4. B, C).

### Aberration and strehl ratio

When the corneal diameter range is 3 mm (Table 2), at each follow-up point after the surgery, both ${Z}_{4}^{0}$ and ${Z}_{6}^{0}$ of the non-dominant eye showed a significant negative shift compared to the measurements taken before the surgery, and the differences were statistically significant(χ^2^ = 50.02, *p*<0.001; χ^2^ = 41.00, *p*<0.001). There were no statistically significant differences when comparing each pair of follow-up points after the surgery. There were no significant changes in ${Z}_{4}^{0}$ and ${Z}_{6}^{0}$ of the dominant eye. The vertical coma (${Z}_{3}^{-1}$) of the non-dominant eye increased significantly in the negative direction before and after the surgery (χ^2^ = 16.04, *p* = 0.003). There were no significant changes among the follow-up points after the surgery.

**Table 2:** Preoperative and postoperative changes in corneal aberrations (Zone=3 mm)

Aberration	Eye	Pre	1w	1mo	3mo	6mo	χ^2^	*p* value
${Z}_{4}^{0}$	DE	0.05(0.03,0.06)	0.03(0.01,0.06)	0.04(0.02,0.06)	0.03(-0.01,0.06)	0.04(0.02,0.06)	3.47	0.482
	NE	0.05 (0.02,0.08)	-0.10 (-0.16,-0.05) ^a^	-0.08 (-0.16,-0.04) ^a^	-0.08 (-0.13,-0.04) ^a^	-0.06 (-0.15,-0.04) ^a^	50.02	<0.001
${Z}_{6}^{0}$	DE	0.00(-0.01,0.00)	0.00(-0.04,0.03)	0.00(-0.03,0.05)	0.00(-0.04,0.02)	-0.06(-0.15,-0.04)	1.42	0.84
	NE	0.00 (-0.04,0.01)	-0.06 (-0.14,-0.02) ^a^	-0.08 (-0.17,-0.04) ^a^	-0.07 (-0.17,-0.03) ^a^	-0.06 (-0.16,-0.02) ^a^	41.00	<0.001
${Z}_{3}^{-1}$	DE	-0.04(-0.07,0.04)	-0.04(-0.10,0.03)	-0.04(-0.10,0.02)	-0.05(-0.12,0.03)	-0.04(-0.12,0.03)	4.519	0.34
	NE	-0.01 (-0.05,0.03)	-0.01 (-0.09,0.03)	-0.03 (-0.09,-0.01) ^a^	-0.06 (-0.13,-0.02) ^a^	-0.06 (-0.10,0.00) ^a^	16.04	0.003
${Z}_{3}^{1}$	DE	-0.01(-0.11,0.07)	-0.11(-0.20,0.01)	-0.08(-0.21,-0.04)	-0.14(-0.20,-0.05)^a^	-0.09(-0.17,-0.05)	15.59	0.004
	NE	0.01(-0.03,0.07)	0.05(-0.02,0.14)	0.04(-0.02,0.15)	0.05(-0.04,0.18)	0.06(-0.03,0.16)	6.389	0.172

DE=dominant eyes. NE=non-dominant eyes. ^a^ Statistically different compared with preoperative value

When the corneal diameter range is 6 mm (Table 3), after the surgery, the ${Z}_{4}^{0}$ of the non-dominant eye increased slightly, and there was no statistically significant difference compared with that before the surgery. The ${Z}_{4}^{0}$ of the dominant eye increased positively, and the difference was statistically significant (χ^2^ = 50.26, *p*<0.001). Compared with the dominant eye, the ${Z}_{4}^{0}$ of the non-dominant eye was more negative, and the difference was statistically significant (*p*<0.001). After the surgery, the ${Z}_{6}^{0}$ of both the dominant eye and the non-dominant eye increased positively, and the differences were statistically significant (*p* < 0.001). There were no significant changes in the horizontal coma (${Z}_{3}^{1}$) of the non-dominant eye and ${Z}_{3}^{-1}$ in dominant eyes.

Both the HOAs of the dominant eye and the non-dominant eye increased positively after the surgery, and the differences were statistically significant (*p* < 0.05). There were no statistically significant differences in the changes of total ocular aberrations (TOAs).

**Table 3:** Preoperative and postoperative changes in corneal aberrations (Zone=6 mm)

Aberration	Eye	Pre	1w	1mo	3mo	6mo	χ^2^	*p* value^◊^
${Z}_{4}^{0}$	DE	0.18(0.15,0.21)	0.35(0.30,0.44)^a^	0.39(0.30,0.42) ^a^	0.38(0.32,0.41) ^a^	0.39(0.35,0.44) ^a^	50.26	<0.001
	NE	0.16(0.13,0.20)	0.08(0.03,0.21)^a^	0.15(0.06,0.22)	0.18(0.08,0.25)	0.17(0.09,0.25)	15.96	0.003
${Z}_{6}^{0}$	DE	0.00(-0.01,0.00)	0.07(0.05,0.11)^a^	0.06(0.05,0.10) ^a^	0.07(0.04,0.11) ^a^	0.06(0.04,0.10) ^a^	43.28	<0.001
	NE	0.00(-0.01,0.01)	0.18(0.13,0.20) ^a^	0.15(0.12,0.21) ^a^	0.14(0.12,0.16) ^a^	0.14(0.11,0.17) ^a^	57.46	<0.001
${Z}_{3}^{-1}$	DE	-0.02(-0.14,0.11)	-0.01(-0.13,0.16)	0.02(-0.18,0.16)	0.00(-0.14,0.15)	-0.01(-0.16,0.13)	2.3	0.681
	NE	-0.03(-0.21,0.12)	-0.10(-0.33,0.06)	-0.05(-0.36,0.06)	-0.13(-0.38,0.06)^a^	-0.05(-0.33,0.10)	11.74	0.019
${Z}_{3}^{1}$	DE	-0.06(-0.17,-0.01)	-0.23(-0.30,-0.03)	-0.24(-0.37,-0.05)	-0.21 (-0.37,-0.05)^b^	-0.22(-0.33,-0.05)	15.61	0.004
	NE	0.06(0.01,0.11) ^c^	0.12(0.01,0.18) ^c^	0.14(-0.01,0.20) ^c^	0.16(0.05,0.20) ^c^	0.19(0.07,0.22)^c^	12.82	0.016
HOAs	DE	0.33(0.29,0.38)	0.57(0.49,0.69) ^a^	0.60(0.46,0.74) ^a^	0.60(0.50,0.70) ^a^	0.63(0.51,0.72) ^a^	45.99	<0.001
	NE	0.33(0.28,0.43)	0.45(0.40,0.53) ^a^	0.44(0.39,0.59) ^a^	0.49(0.39,0.56) ^a^	0.49(0.38,0.57) ^a^	28.37	<0.001
TOAs	DE	0.80(0.48,1.08)	0.86(0.68,0.98)	0.80(0.66,0.99)	0.88(0.67,0.97)	0.81(0.67,1.00)	0.77	0.943
	NE	0.85(0.60,1.25)	0.81(0.55,0.94)	0.74(0.68,0.89)	0.79(0.63,0.94)	0.77(0.64,0.97)	7.31	0.12

DE=dominant eyes. NE = non-dominant eyes. ^a^ Statistically different compared with preoperative value. ^b^ Statistically different between 1 month and 3month after surgery. ^c^ After adjusting the significance value using the Bonferroni correction method, there was no statistically significant difference between the various time points. ^d^ Statistically different between 1 week and 6 month after surgery

The SR demonstrated no significant changes postoperatively compared to preoperative levels within both the 3-mm and 6-mm zones (*p* > 0.05). The change of SR over time is shown in Table 4.

**Table 4:** The change of SR

Parameter	eye	pre	1w	1mo	3mo	6mo	*p* value
Zone=3 mm							
SR	DE	0.3479 (0.2302,0.4623)	0.3831 (0.3090,0.4388)	0.3928 (0.2928,0.4973)	0.3722 (0.2788,0.4607)	0.3478 (0.2713,0.5191)	0.99
	NE	0.2586 (0.2069,0.4062)	0.3202 (0.2585,0.3854)	0.3129 (0.2628,0.3797)	0.3141 (0.2569,0.3784)	0.3044 (0.2637,0.3841)	0.63
Zone=6 mm							
SR	DE	0.1328 (0.0953,0.2020)	0.1498 (0.1150,0.1835)	0.1540 (0.1105,0.1912)	0.1338 (0.0984,0.1866)	0.1595 (0.1259,0.2244)	0.47
	NE	0.1340 (0.0861,0.1800)	0.1199 (0.0902,0.1509)	0.1146 (0.1030,0.1489)	0.1097 (0.0958,0.1459)	0.1208 (0.1064,0.1377)	0.6

Patient Satisfaction and Spectacle Independence Rate

The score of the NEI-VFQ-25 increased from 74.63 (71.70, 81.81) before the operation to 91.15 (85.23, 93.08) after the operation (*p* < 0.001), and the satisfaction with visual function was significantly improved, especially the near visual acuity. The spectacle independence rate reached 96% at 6 months after the operation. Only one patient needed to wear glasses for distance vision when driving at night.

## Discussion

Presbyopia is a phenomenon in which the ability to accommodate decreases due to age-related factors, leading to difficulties in near vision. Although this is a normal physiological phenomenon, it still causes trouble for patients with presbyopia [1]. In recent years, with the increasing demand for presbyopia patients to get rid of glasses, refractive surgery for correcting presbyopia has become a “Holy Grail”. The correction of presbyopia can be divided into static correction and dynamic correction. Currently, dynamic correction (including accommodating intraocular lenses, scleral expansion, etc.) still has difficulty completely restoring patients’ dynamic accommodation ability. Therefore, static correction, especially refractive surgery for correcting presbyopia, remains the mainstream method [3].

PresbyLASIK employs laser in-situ keratomileusis (LASIK) to reshape the cornea into a multifocal configuration for correcting refractive errors accompanied by presbyopia. Depending on the multifocal partition design, PresbyLASIK can be categorized into two types: the central type (which provides near vision in the central zone and distance vision in the periphery) and the peripheral type (which offers near vision in the peripheral zone and distance vision centrally) [9]. In this study, we employed PresbyMAX, a central-type PresbyLASIK technique. This approach integrates the benefits of micro-monovision, multifocality, and bi-asphericity, reducing interocular anisometropia while reshaping the cornea into an aspheric profile with a steeper central region and a flatter peripheral region. By incorporating DoF, this approach enhances near vision while mitigating the visual discomfort associated with anisometropia. Based on variations in the DoF and anisometropia, PresbyMAX can be categorized into four treatment modalities: symmetric, hybrid, µ-monovision, and the monocular scheme (employed in this study). Previous studies have primarily focused on the first three correction modalities. In this investigation, we evaluated visual outcomes following PresbyMAX monocular treatment for myopia with concomitant presbyopia by analyzing distance, intermediate, and near visual acuity along with optical aberrations at six months postoperatively. Furthermore, surgical efficacy was assessed through comparative analysis of pre- versus postoperative Strehl ratios and visual quality indices.

Early studies indicate that while symmetric correction mode—which employs bilateral symmetrical treatment—effectively enhances near vision, its efficacy in improving distance vision remains suboptimal, likely due to reduced interocular contrast sensitivity [12]. Luger et al. [13] reported that hybrid mode correction for hyperopia and myopia with concomitant presbyopia resulted in over 90% of patients achieving BUDVA of 20/20 or better and BUIVA of J2 or better. Notably, 93% of myopic patients and 88% of hyperopic patients attained a BUNVA of J2 or better postoperatively. Kohnen et al. [14] compared the efficacy of hybrid and µ-monovision schemes for correcting myopia with presbyopia, reporting that both modalities significantly enhanced distance, intermediate, and near visual acuity. However, no statistically significant difference in uncorrected visual acuity (UCVA) outcomes was observed between the two approaches.

Comparative analysis of these studies reveals that multifocal ablation achieves significant near vision gains but partially compromises distance vision outcomes. In contrast, hybrid and µ-monovision approaches strategically limit the DoF introduction in the dominant eye, thereby enhancing postoperative contrast sensitivity and intermediate visual acuity. Nevertheless, the bilateral application of multifocal ablation, coupled with induced negative spherical aberration, may necessitate a neuroadaptive period for patients to achieve optimal visual integration [15]. Our findings indicate significant improvements in distance, intermediate, and near visual acuity outcomes at six months postoperatively compared to preoperative baselines. These gains appeared to plateau between the 3‑ and 6‑month postoperative visits, suggesting a sustained treatment effect. At 6 months postoperatively, 96% of patients achieved a BUDVA of 0.1 logMAR or better. These outcomes are largely consistent with the findings of Fu et al. [16], lending further support to the efficacy of this procedure in presbyopia management. Furthermore, comparison with prior studies suggests that the PresbyMAX monocular approach may offer a potential advantage in enhancing distance vision outcomes. Preliminary data suggested that patients achieved functional distance vision early postoperatively, potentially requiring a shorter neuroadaptation period than with bilateral multifocal strategies. Consequently, PresbyMAX monocular may be particularly suitable for individuals requiring rapid recovery of sharp distance vision. Consequently, the PresbyMAX monocular modality could represent a valuable option for patients who need rapid recovery of sharp distance vision. At the 6-month postoperative assessment, all patients achieved a UIVA of 0.1 logRAD or better in the dominant eye, and 92% of patients had UIVA of 0 log RAD or better; 84% of non-dominant eyes had a UIVA of 0 log RAD or better. By comparing the binocular vision results and patients’ subjective feelings, it was found that the dominant eye plays a major role when viewing medium-distance objects. Before surgery, only 28% of eyes achieved a UNVA of J3, while at 6 months after surgery, this proportion increased to 100%, and 96% of patients even achieved a BUNVA of J1 or better. Based on our observations, the monocular approach may not only preserve better distance vision but also attain similarly favorable near visual outcomes compared to the conventional bilateral multifocal PresbyMAX technique.

Comparative analysis of preoperative and postoperative CDVA revealed favorable safety outcomes. At three months postoperatively, only one eye exhibited a one-line loss in CDVA, with no instances of two-line or greater decline. By six months, no CDVA loss was observed across the cohort. Notably, CDVA improved by two lines in 4% of eyes, one line in 42%, and remained stable in 48% of eyes compared to preoperative baselines—underscoring the procedure’s safety profile. These findings align with the safety outcomes reported by Chan et al. [17], validating the technique’s safety.

Wavefront aberration, defined as the optical discrepancy between an ideal and the actual retinal image, represents a critical determinant of visual quality [18]. These aberrations degrade retinal image fidelity by transforming focused points into blurred patterns. Paradoxically, this same phenomenon contributes to the DoF, enabling the human eye to perceive objects clearly across a range of distances—a mechanism central to presbyopia compensation. While low-order aberrations (e.g., defocus, astigmatism) are addressable with conventional ophthalmic corrections, high-order aberrations (e.g., coma, spherical aberration) remain uncorrectable by standard spectacles or most contact lenses, necessitating advanced wavefront-guided interventions. Among high-order aberrations, SA exerts the most pronounced influence on central visual acuity and optical quality along the visual axis. In the human eye, SA arises primarily from two sources: corneal SA and lens SA. This aberration can manifest as either positive or negative. Positive SA occurs when peripheral rays converge anterior to the focal point of paraxial rays, whereas negative SA is characterized by peripheral rays focusing posterior to the paraxial focal point [19]. The interplay of these aberrations critically modulates retinal image quality and DoF, with implications for both natural vision and refractive surgical outcomes. Studies demonstrate that during near-vision accommodation, the human eye undergoes characteristic shifts in ${Z}_{3}^{-1}$ and ${Z}_{4}^{0}$ exhibit a negative shift, while ${Z}_{6}^{0}$ demonstrates a mild positive increase. This aberration profile enhances near-visual performance by modulating optical phase gradients [20]. Contemporary presbyopia-correcting interventions strategically modulate aberrations to optimize DoF [21]. While isolated induction of ${Z}_{4}^{0}$ or ${Z}_{6}^{0}$ exerts minimal impact on DoF enhancement, combined induction of negative ${Z}_{4}^{0}$ and positive ${Z}_{6}^{0}$ synergistically maximizes DoF expansion, particularly within the central 0–2 mm pupillary zone. Conversely, co-induction of these aberrations with identical polarities shifts DoF effects predominantly to the peripheral pupillary region [22]. Pupil dynamics further critically modulate DoF efficacy: Under photopic conditions (3 mm pupil diameter), DoF is significantly greater than under mesopic dilation (6 mm), highlighting the importance of neuro-adaptive pupil responses in presbyopia management [23].

Postoperative wavefront analysis in this study revealed distinct aberration profiles between eyes. Within the 3 mm corneal zone, the non-dominant eye exhibited a negative shift in ${Z}_{4}^{0}$ and ${Z}_{6}^{0}$,while the dominant eye showed minimal aberration changes. At the 6 mm corneal diameter, the non-dominant eye demonstrated significantly more negative ${Z}_{4}^{0}$ compared to the dominant eye, coupled with a marked increase in ${Z}_{6}^{0}$.

During near-vision accommodation, pupil constriction modulates optical aberrations in the non-dominant eye. Within the 3-mm corneal zone, both ${Z}_{4}^{0}$ and ${Z}_{6}^{0}$ exhibit a negative shift, resulting in anterior convergence of central light rays relative to peripheral rays—a mechanism that substantially expands the DoF. Notably, ${Z}_{4}^{0}$ demonstrated a more pronounced negative shift in the 3 mm zone compared to the 6 mm corneal region, which may preferentially enhance near-visual performance in the central pupillary area.

While this study observed a marginal increase in ${Z}_{4}^{0}$ 6 mm optical zone postoperatively, the change lacked statistical significance compared to preoperative baselines. However, the non-dominant eye exhibited a significantly more negative ${Z}_{4}^{0}$ profile than the dominant eye, contributing to near vision enhancement. Concurrently, the positive shift in ${Z}_{6}^{0}$ further optimized near vision within the central pupillary zone.

Notably, while controlled induction of negative SA improves presbyopic near vision, excessive aberration can degrade retinal image quality and reduce high-spatial-frequency contrast sensitivity. Thus, optimal outcomes require balancing SA modulation to DoF while preserving optical quality—a principle supported by prior studies [24].

Interestingly, our findings diverge from Ryu et al. [10], who reported a negative ${Z}_{4}^{0}$ shift in both eyes at 6 months postoperatively, with greater values in the dominant eye. In contrast, our cohort showed a nonsignificant ${Z}_{4}^{0}$ increase from 0.16(0.13 ~ 0.20)µm preoperatively to 0.17(0.09 ~ 0.25) µm postoperatively. This discrepancy may stem from differences in preoperative spherical equivalent. During high myopic correction, the inherent positive SA from corneal flattening could neutralize the negative aberration intentionally introduced by the procedure, thereby accounting for the observed stability in ${Z}_{4}^{0}$.

Coma, an HOA characterized by a comet-like spread of light (with a bright central core and fading tail) when rays traverse an optical system, arises from asymmetric refractive deviations across the pupil [19]. Quantified by Zernike coefficients ${Z}_{3}^{-1}$ and ${Z}_{3}^{1}$, this aberration reflects optical skewness and degrades retinal image quality. While ${Z}_{3}^{-1}$ may modestly enhance DoF, ${Z}_{3}^{1}$ induces optical interference, significantly impairing contrast sensitivity and visual quality [25]. In this study, at the 6-month follow-up, no statistically significant changes in ${Z}_{3}^{-1}$ or ${Z}_{3}^{1}$ were observed in the non-dominant eye across 6 mm corneal zones, consistent with the stable coma profiles reported by Fu et al. [21]. However, a subtle negative shift in ${Z}_{3}^{-1}$ was noted within the 3 mm corneal zone, a directional trend that may support enhanced central near-vision performance by marginally expanding the DoF in the pupillary core, and no statistically significant changes in ${Z}_{3}^{1}$. This stability underscores the precision of PresbyMAX monocular ablation in preserving innate corneal asymmetry while mitigating surgically induced coma—a key advantage over conventional techniques. The integration of seven-dimensional (7D) eye-tracking technology further minimizes coma induction, enhancing near vision without compromising optical quality—a critical advance in presbyopia correction.

To quantify visual outcomes, we evaluated the SR, defined as the area under the modulation transfer function (MTF) curve. A higher SR correlates with superior optical quality and aligns robustly with patient-reported visual satisfaction [26, 27]. Our findings demonstrate that the strategic reduction of coma, coupled with optimized SA profiles, preserves SR values postoperatively, validating the procedure’s efficacy in balancing functional gains with optical fidelity. The stability of the Strehl ratio observed in this study indicates that the surgical procedure did not adversely affect the objective optical quality as measured by this parameter (Table 4). These findings contrast with the outcomes reported by Luger et al. [13], where the Hybrid mode for correcting refractive errors with concomitant presbyopia resulted in a significant postoperative reduction in SR, a metric reflecting degraded optical quality. This disparity underscores the inherent trade-offs of bilateral multifocal strategies: while effective for expanding DoF, they introduce competing aberrations that cumulatively impair retinal image fidelity. Our findings suggest that the PresbyMAX monocular approach appeared to reduce such trade-offs by restricting multifocal modulation to the non-dominant eye, which was associated with preserved SR stability alongside comparable near-vision gains. The observed negative shift in ${Z}_{3}^{-1}$ within the 3 mm corneal zone of the non-dominant eye further supports its role in enhancing near vision by expanding the DoF. This directional aberration modulation aligns with presbyopia correction principles, where controlled induction of asymmetric optical profiles optimizes functional outcomes. However, the clinical utility of selectively introducing ${Z}_{3}^{-1}$—akin to the strategic application of negative SA—remains underexplored. While our findings demonstrate that such coma shifts may improve near vision without degrading overall optical quality, deliberate coma induction requires further investigation to balance its benefits against potential dysphotopsia risks.

Postoperative analysis revealed increased HOAs in both eyes, consistent with prior studies of multifocal ablations. Crucially, however, TOAs remained stable, indicating that the procedure did not introduce significant additional aberrations beyond the intended multifocal profile. This stability, corroborated by Fu et al. [21], underscores the precision of modern wavefront-guided platforms in minimizing induction of unintended aberrations.

We assessed preoperative and postoperative visual function using the NEI-VFQ-25, with a focus on near-vision performance. Median visual function scores improved significantly from 74.63 (71.70–81.81) preoperatively to 91.15 (85.23–93.08) postoperatively, reflecting enhanced subjective visual satisfaction. Additionally, the spectacle independence rate reached 96%, with only one patient requiring myopic correction for nighttime driving. All other patients reported no need for glasses in daily activities. These outcomes contrast with Luger et al. [13], where bilateral Hybrid mode correction (multifocal ablation in both eyes) resulted in reduced postoperative visual quality due to dysphotopsia (e.g., diplopia, ghosting, halos), despite improved near vision. This disparity likely stems from the inherent trade-offs of bilateral multifocal ablation, which amplifies interocular interference. In contrast, our findings align with Fu et al. [16], who similarly employed a monocular correction strategy and observed high patient satisfaction with minimal visual disturbances, underscoring the advantage of asymmetric presbyopia management.

The primary limitation of this study is its small sample size, attributable to patient hesitancy toward novel presbyopia correction methods. Future research should prioritize comparative trials evaluating PresbyMAX against other presbyopia-correction modalities (e.g., Hybrid, µ-monovision) to identify optimal candidates for each approach. Additionally, long-term studies are warranted to assess neuroadaptation dynamics and the sustainability of visual outcomes. The assessment of optical quality using the SR alone was insufficient to fully represent functional vision. To address this limitation in future investigations, we plan to include contrast sensitivity testing to explore the correlation between theoretical aberrations and clinical performance.

The PresbyMAX monocular mode is a safe and effective strategy for presbyopia correction, selectively inducing negative spherical aberration to expand DoF without compromising optical quality. This approach yielded significant improvements in full-range visual acuity, with 96% spectacle independence and high patient satisfaction at 6 months postoperatively. It is particularly suitable for presbyopic individuals prioritizing distance vision, offering a balanced solution for all-distance visual clarity.

## Data Availability

The datasets used and/or analysed during the current study are available from the corresponding author on reasonable request.

## Abbreviation

UDVA: Uncorrected Distance Visual Acuity
UIVA: Uncorrected Intermediate Visual Acuity
UNVA: Uncorrected Near Visual Acuity
CDVA: Corrected Distance Visual Acuity
SR: Strehl Ratio
NEI-VFQ-25: National Eye Institute 25-Item Visual Function Questionnaire
logMAR: Logarithm of the Minimum Angle of Resolution
logRAD: Logarithm of the Reading Acuity Determination
D: Diopter
CNVA: Corrected Near Visual Acuity
DE: Dominant Eye
NE: Non-Dominant Eye
SE: Spherical Equivalent
ADD: Addition (for near vision)
BUIVA: Binocular Uncorrected Intermediate Visual Acuity
SA: Spherical Aberration
HOA: Higher-Order Aberrations
DoF: Depth of Focus
LASIK: Laser In-Situ Keratomileusis
UCVA: Uncorrected Visual Acuity
MTF: Modulation Transfer Function
TOA: Total Ocular Aberrations
